# The "Universal Review" and Our Critics

**Published:** 1888-06-23

**Authors:** 


### The "Universal Review" and our Critics.

The June number of the *Universal Review* contains an article entitled "The College of Physicians and the Medical Press," which deals very sharply, not only with that body, but with the journal which always gives itself out—with what exactitude we do not care to say—as the organ of the profession, the *Lancet*. That the *Lancet* was this once neither we nor Mr. Edward Berdoe, the writer of the article in question, care to deny; but, as he says, it is "a periodical which a literary evolutionist could easily show to have undergone a 'transformation' and 'adaptation' of a very remarkable character. . . . From a professional organ written by doctors for doctors, dealing with strictly professional and technical matters for professional purposes, from an organ of medical science intended for the use of adepts in that department of learning, the *Lancet* has so far yielded to the influences of its surroundings and interests as to have degenerated into a vehicle for conveying medical information, not always of a very desirable sort, to the general public, Indeed it is matter of common knowledge that the *Lancet* finds its widest public outside the circle of the profession for which ostensibly it is designed."

This is rather a serious accusation, especially in view of the indubitable truth of the description Mr. Berdoe gives of the *Lancet's* composition. Here it is, detailed in the fashion of the cookery book: "Take of advertisements about anything in the heavens above, the earth beneath, or the waters under the earth, which can possibly interest a more or less imaginary invalid—seventy pages; of a pleasant discourse on embryonic life, highly interesting and instructive to our young people—two pages and a half; of downright heavy and profoundly scientific stuff, interesting to nobody but the writers—some dozen pages; of accounts of special operations, interesting only to those who are likely to have cases requiring such treatment, and therefore mere advertisements for the business carried on by the operators—half-a-score of pages; next, some twenty columns of coloured and pseudo-scientific paragraphs about subjects connected with our homes, our amusements, and our occupations; a little about our Queen, and a good deal about our neighbour's Kaiser; many columns about our taxes, our hard times, the climates we might enjoy, the sort of weather that torments us." And this claims to be the organ of the profession! Say, rather, the advertising medium of the profession!

For this is the point of it all: that anyone who so chooses may buy the *Lancet*. It is not, like the *British Medical Journal*, the leader of medical opinion, and the organ of a scientific association with a circulation confined as much as may be to members of the medical profession; it is, as Mr. Berdoe points out, to be bought at every railway bookstall by all and sundry of either sex who choose to give the price demanded for it. All and sundry men and women do buy it and read it, with what results to their inner consciousness need not be said; for there are many matters, physiological and pathological, which the scientist studies simply and even reverently, as facts of nature, normal or abnormal, which in the untrained or pruriently curious mind raise the most unwholesame emotions. "To tout for the general reader," says Mr. Berdoe, "who can have no just concern with the greater part of the information supplied him, is a gross offence against public decency."

All this has long been a matter of general knowledge, it has of late been a matter of general talk, but the College of Physicians, while censuring the publication by medical men of signed articles in journals where the proprieties of civilised life are more carefully preserved than in any of our daily papers, has excepted the *Lancet* from its censure. Why? Because—so at least most people believe, and Mr. Berdoe states—many members of that august body have a commercial interest in having their names thus brought before the general public. This has been whispered before now among members of the profession; but Mr. Berdoe is the first medical man who has had the manly courage to say so openly in a public journal.

